# Supplementary material for: A modelled evaluation of the impact of COVID-19 on breast, bowel, and cervical cancer screening programmes in Australia
Source: eLife. 2023 Apr 6;12:e82818. doi: 10.7554/eLife.82818 (PMC10079286; doi:10.7554/eLife.82818)
Supplement: Supplementary file 1. — (A) Ranking of women aged 50–74 in the queue for available screens during the recovery period. (B) Proportion of eligible women with any Cervical Screening Test recorded – modelled assumption prior to disruption compared to observed data (1 December 2017–14 January 2020), by age. (C) Routine and follow-up attendance assumptions for counterfactual (no-disruption) and disruption scenarios to the NCSP. (D) Selected estimated outcomes by 2-y calendar period (2020–2021), 2021–2022, and 2022–2023 assuming a 12-mo disruption, for women aged 50–74. (E) Estimated screening delays and participant profiles for each BreastScreen programme disruption scenarios modelled, reported for various calendar periods. (F) Diagnostic assessments and short-term adenoma outcomes. (G) Impact of disruption on cancer outcomes among women aged 25–74 y, 2020–2022. (H) Impact of disruption on number of women expected to attend for an HPV test (any purpose). (I) Impact of disruption on expected colposcopy utilisation, 2020–2022. [file elife-82818-supp1.docx]

**Supplementary File 1A**

**Ranking of women aged 50-74 in the queue for available screens during the recovery period.**

| **Client group** | **Rank in queue** | **Target criteria** |
| --- | --- | --- |
| A. New BreastScreen clients who become eligible for screening (i.e., reach age 50) during *screening pause*; age range 50-74. | Rank = 1, also ensuring criteria is met | Screened at a maximum of 15 months later than would have been originally scheduled. |
| B. New BreastScreen clients who become eligible for screening (i.e., reach age 50) during *screening recovery*; age range 50-74. | Rank = 2, also ensuring criteria is met | Screened at a maximum of 15 months later than would have been originally scheduled. |
| C. Existing BreastScreen clients who would have been scheduled for screening during *screening pause*; age range 50-74. | Rank = 3, also ensuring criteria is met | Screened at a maximum of 21 months later than originally scheduled. |
| D. Existing BreastScreen clients who become eligible for screening (i.e., reach age 50) during *screening recovery*; age range 50-74. | Rank = 4, also ensuring criteria is met | Screened at a maximum of 21 months later than originally scheduled. |

The prioritisation module prioritised clients during the recovery period and assigned them to available screens following screening resumption. Available screens were assigned as follows:

i) For each week of the recovery period, clients queued for screening (either carried over from the pause or due under status quo during the recovery period) were prioritised for available screening appointments according to their screening round (first or subsequent), age, and whether their screen was due or overdue, as shown in Table 2. Women who usually screen annually were indirectly prioritised. The queue included only women aged 50-74 years.

ii) The module aimed to firstly assign clients at the top of the queue to available screening appointments for that week, and then push the remaining queue forward week by week until they were able to be assigned to an available appointment. This pushing forward was limited by a threshold using the maximum delays as described in Table2.

iii) Clients resumed normal screening schedules after their first recovery screen.

**Supplementary File 1B**

**Proportion of eligible women with any Cervical Screening Test recorded – modelled assumption prior to disruption compared to observed data (1 Dec 2017-14 Jan 2020), by age**

| **Age group** | **Model assumption (% attended prior to disruption** | **Observed data (1 Dec 2017-14 Jan 2020)** |
| --- | --- | --- |
| 25–29 | 39.3 | 39.0 |
| 30–34 | 48.5 | 49.7 |
| 35–39 | 55.4 | 54.6 |
| 40–44 | 57.7 | 58.1 |
| 45–49 | 59.9 | 60.7 |
| 50–54 | 62.6 | 61.5 |
| 55–59 | 61.4 | 61.6 |
| 60–64 | 60.5 | 60.6 |
| 65–69 | 58.0 | 58.1 |
| 70–74 | 32.0 | 35.2 |

**Supplementary File 1C**

**Routine and follow-up attendance assumptions for counterfactual (no-disruption) and disruption scenarios to the NCSP.**

| **Scenario** | **Routine screening attendance assumptions ^a^** | **Follow-up attendance assumptions ^b^** |
| --- | --- | --- |
| Status quo – no disruption | No disruption to routine attendance in 2020. | No disruption to follow-up in 2020. |
| Disruption scenarios (see Table 1) | Screening disruption reflected as a percent decrease in attendance across 2020 ^c^. | Women who are HPV not 16/18 positive with LBC <= LSIL receive 12-month follow-up. If this follow-up occurred in 2020, and they were still HPV not 16/18 positive with LBC <= LSIL, they were recommended to return for further follow-up in an additional 12 months instead of being referred for colposcopy (under pre-COVID management). If at this third test they are still any HPV positive, they will be referred to colposcopy. Management of HPV negative, HPV 16/18 positive and HPV not 16/18 positive women with LBC >= HSIL was unchanged. This change to the 2017 Guidelines (40) was assumed to be temporary and in place for the period of COVID-19 disruption only. |

^a^ Majority of routine screening attenders previously screened with cytology 2+ years prior, with 38.4-64.8% of eligible women failing to attend for their first HPV test (NCSR data; see Data sources www.policy1.org/models/cervix). Re-attendance after 2+ years reflects pre-renewed NCSP data indicating that women are likely to re-attend by 3-5 years. ^b^ No changes in follow-up management or attendance are assumed for women already under follow-up management. ^c^ As the disruption in practise occurred from ~late March 2020, rather than at the beginning of the year, model predictions for 2020 correspond to approximately late March 2020 – late March 2021, and so on for later years.

**Supplementary File 1D**

**Selected estimated outcomes by two-year calendar period (2020-2021), 2021-2022 and 2022-2023 assuming a 12-month disruption, for women aged 50-74. Proportional changes compared to status quo are shown in brackets. For all scenarios, screening is assumed to resume gradually after the pause to services, reaching status quo rates by the seventh month after resumption.**

| **Outcome** | **Status quo** | | | **12-month pause** | | |
| --- | --- | --- | --- | --- | --- | --- |
|  | **2020-2021** | **2021-2022** | **2022-2023** | **2020-2021** | **2021-2022** | **2022-2023** |
| Invasive breast cancers per 100,000 women^a^ | 298 | 298 | 299 | 270 (-9%) | 300 (1%) | 296 (-1%) |
| Screen-detected invasive breast cancers per 100,000 women^a^ | 127 | 123 | 122 | 97 (-24%) | 127 (3% | 119 (-2%) |
| Interval cancers (12 month)^b^ | 15 | 15 | 15 | 8 (-47%) | 10 (-33%) | 13 (-13%) |
| Interval cancers (27 month)^b^ | 38 | 39 | 38 | 33 (-13%) | 26 (-33%) | 30 (-21%) |
| Program sensitivity^c^ | 76.8% | 76.2% | 76.4% | 74.8% (-3%) | 82.5% (8%) | 79.9% (5%) |
| Tumour size (% ≤15mm diameter) | 59.7% | 59.3% | 59.3% | 56.5% (-5%) | 57.0% (-4%) | 57.0% (-4%) |
| Nodal involvement (% involving nodes) | 24.9% | 25.2% | 25.5% | 26.4% (6%) | 26.9% (7%) | 26.7% (5%) |
| Grade (% Grade 3 versus Grade 1/2) | 46.6% | 46.1% | 45.8% | 48.4% (4%) | 46.9% (2%) | 47.4% (3%) |
| Recall rate (N)^c^ | 5.2% | 5.2% | 5.2% | 5.6% (8%) | 5.6% (8%) | 5.4% (4%) |
| False positive recall rate (N)^d^ | 4.6% | 4.6% | 4.6% | 4.8% (4%) | 4.9% (7%) | 4.7% (2%) |

1. Rates are per 100,000 women in the Australian population, including women who do not usually participate in screening.
2. Invasive breast cancers arising within 12 or 27 months of a negative screening episode.
3. The proportion of screening episodes requiring recall for further investigation
4. The proportion of screening episodes requiring recall for further investigation, with a benign final outcome after that investigation.

**Supplementary File 1E**

**Estimated screening delays and participant profiles for each BreastScreen program disruption scenarios modelled, reported for various calendar periods. Figures in brackets describe the proportional difference of screening intervals to status-quo median screening intervals of 104 weeks (e.g. 27% means a 27% longer median screening interval).**

| **Outcome** | **Duration of pause** | **Client group*** | | | |
| --- | --- | --- | --- | --- | --- |
|  |  | **Directly affected** | **Indirectly affected** | **Not affected** | **Total** |
| Screening client profile, 50-74, 2020-2021 | No pause | N/A | N/A | 100% | 100% |
|  | 3 months | 14% | 11% | 75% | 100% |
|  | 6 months | 23% | 29% | 48% | 100% |
|  | 12 months | 54% | 27% | 19% | 100% |
| Screening client profile, 50-74, 2022-2023 | No pause | N/A | N/A | 104 | 100% |
|  | 3 months | 11% | 10% | 79% | 100% |
|  | 6 months | 21% | 25% | 54% | 100% |
|  | 12 months | 37% | 49% | 14% | 100% |
| Screening interval (median weeks) 2020-2021 | No pause | N/A | N/A | 104 | 104 |
|  | 3 months | 132 (27%) | 126 (21%) | 104 (0%) | 107 (3%) |
|  | 6 months | 151 (45%) | 133 (28%) | 104 (0%) | 113 (9%) |
|  | 12 months | 154 (48%) | 114 (10%) | 103 (-1%) | 130 (25%) |

* Directly affected: BreastScreen clients whose screens were originally scheduled for the pause period; indirectly affected: BreastScreen clients whose screens were originally scheduled after the pause period and whose screens are delayed during the recovery period to help accommodate directly affected women; not affected: BreastScreen clients with no delay to their screens but with screens or outcomes within the reporting periods.

Note: time between screens combines modelled intervals for women who missed screens in the pause and newly-due women according to prioritisation described earlier.

**Supplementary File 1F**

**Diagnostic assessments and short-term adenoma outcomes**

| **Outcomes** | **Comparator (no pause)** | **12 month pause** | **6 month pause** | **3 month pause** |
| --- | --- | --- | --- | --- |
| Screening follow-up colonoscopies, 2020-2021 | 149,035 | 74,610 | 111,815 | 130,388 |
| decrease (%) in 2020-2021 | - | 74,425 (49.94%) | 37,220 (24.97%) | 18,647 (12.51%) |
| NBCSP surveillance colonoscopies, 2020-2021 | 45,920 | 44,219 | 44,804 | 45,416 |
| decrease (%) in 2020-2021 | - | 1,701 (3.7%) | 1,115 (2.43%) | 504 (1.1%) |
| Adenomas not detected at screening, 2020-2021* | - | 31,811 | 15,827 | 7,917 |
| Advanced adenomas not detected at screening, 2020-2021* | - | 17,156 | 8,536 | 4,270 |

* This outcome is an estimate of the number of adenomas (including advanced and non-advanced adenomas) and advanced adenomas which would have been detected at NBCSP follow-up screening in 2020 or 2021 without any pause to screening, but would be missed with the corresponding pause to screening.

**Supplementary File 1G**

**Impact of disruption on cancer outcomes among women aged 25-74 years, 2020-2022**

| Scenario | **Additional cancers*** | | **Cancers upstaged** | | **Additional deaths due to additional/ upstaged cancers over 2020-2022** |
| --- | --- | --- | --- | --- | --- |
|  | **N** | **% increase** | **Localised → Regional** | **Regional → distant** |  |
| No disruption | 1,878 | n/a | n/a | n/a | n/a |
| S1: 12m 95% ↓ | 69 | 3.6% | 18 | 9 | 20 |
| S2: 9m 75% ↓ | 34 | 1.8% | 8 | 4 | 9 |
| S3: 6m 50% ↓ | 21 | 1.1% | 6 | 3 | 6 |

Includes outcomes in women aged up to 74 years in 2020, who will be aged up to 76 in 2022. Cancer deaths includes deaths that would be expected to occur outside 2020-2022 but are a result of delayed diagnosis due to the disruption in 2020.

* No disruption scenario row shows number expected in that scenario; other rows show difference in relation to the no disruption scenario. Upstaged localised cancers that progressed to regional were calculated by assuming that the number of upstaged cancers is equal to the difference in numbers of diagnosed regional cancers, corrected for the number of regional cancers which were upstaged to distant. Upstaged regional cancers that progressed to distant were calculated by assuming that the number of upstaged cancers is equal to the difference in numbers of diagnosed distant cancers, corrected for the number of distant cancers which resulted in cancer death in the simulated period. Some differences between Tables are due to rounding cases to whole numbers.

**Supplementary File 1H**

**Impact of disruption on number of women expected to attend for an HPVtest (any purpose)**

| Scenario | **Women screened/ who missed screening*, 2020** | | **Additional women screened** | |
| --- | --- | --- | --- | --- |
|  | **N** | **%** | **2021** | **2022** |
| No disruption | 1,413,888 |  | n/a | n/a |
| S1: 12m 95% ↓ | 1,027,437 | 72.7% | 407,077 | 479,226 |
| S2: 9m 75% ↓ | 608,351 | 43.0% | 542,670 | 7,808 |
| S3: 6m 50% ↓ | 270,378 | 19.1% | 103,318 | 123,982 |

Includes women with an HPV test for any purpose, including follow-up tests or tests in symptomatic women that result in a cancer diagnosis, but excludes women with an HPV test for symptoms who are not diagnosed with cervical cancer. * No disruption scenario row shows number expected in that scenario; other rows show difference in relation to the no disruption scenario.

**Supplementary File 1I**

**Impact of disruption on expected colposcopy utilisation, 2020-2022***

| Scenario | **Difference in number (%) of colposcopies** | | | | | | **Cumulative (2020-2022)** | |
| --- | --- | --- | --- | --- | --- | --- | --- | --- |
|  | **2020** | **%** | **2021** | **%** | **2022** | **%** | **N** | **%** |
| No disruption** | 101,376 | n/a | 79,476 | n/a | 64,768 | n/a | 245,620 | n/a |
| S1: 12m 95% ↓ | -47,868 | -47.2% | -3,633 | -4.6% | 17,325 | 26.8% | -34,175 | -13.9% |
| S2: 9m 75% ↓ | -32,208 | -31.8% | 7,947 | 10.0% | 9,024 | 13.9% | -15,237 | -6.2% |
| S3: 6m 50% ↓ | -17,680 | -17.4% | 527 | 0.7% | 4,995 | 7.7% | -12,157 | -4.9% |

Excludes colposcopies in women with an HPV test for symptoms who are not diagnosed with cervical cancer. These estimates do not explicitly take into account colposcopy capacity or waiting lists (this information was not available) but do incorporate less than perfect adherence to colposcopy. The disruptions modelled are predicted to result in around 12,157-34,175 fewer colposcopies over 2020-2022.

** No disruption scenario row shows number expected in that scenario; other rows show difference in relation to the no disruption scenario.
